# Pitch as a Shared Code for Music and Speech: Behavioral and Neural Evidence From Stroke Patients

**DOI:** 10.1002/brb3.71512

**Published:** 2026-05-29

**Authors:** Aleksi J. Sihvonen, Tommi Makkonen, Teppo Särkämö

**Affiliations:** ^1^ Cognitive Brain Research Unit (CBRU), Department of Psychology University of Helsinki Helsinki Finland; ^2^ Centre of Excellence in Music, Mind, Body and Brain University of Helsinki Helsinki Finland; ^3^ Department of Neurology Helsinki University Hospital Helsinki Finland; ^4^ Queensland Aphasia Research Centre University of Queensland Brisbane Australia; ^5^ School of Health and Rehabilitation Sciences University of Queensland Brisbane Australia

**Keywords:** amusia, connectivity, music, pitch, prosody, stroke

## Abstract

**Introduction:**

Accurate pitch processing, including the ability to discriminate small differences in pitch, is essential for interpreting the melodic structure of both music and speech. However, the extent to which pitch discrimination is a shared auditory‐perceptual processing component in these domains remains unclear.

**Methods:**

This clinical study explored pitch discrimination, musical pitch perception, and linguistic and affective prosody perception in 39 stroke patients. Behavioral testing was performed during both early subacute (within 3 weeks of stroke) and late subacute (3 months post‐stroke) stages, and whole‑brain white matter connectome analyses of early acute stage diffusion MRI data were conducted to identify shared neural substrates of these auditory functions. In addition, prognostic analyses were carried out examining whether early subacute white matter integrity predicted late subacute pitch‑related outcomes.

**Results:**

Across both stages, pitch discrimination showed robust associations with musical pitch perception and both linguistic and affective prosody perception. Deviation‐specific analyses indicated that the 50‐cent pitch discrimination threshold was the most sensitive index of individual differences, showing the strongest and most consistent associations with musical and prosodic abilities. Early subacute cross‐sectional connectome analyses revealed overlapping neural substrates: better performance across all four auditory domains was linked to greater integrity of the right inferior fronto‑occipital fasciculus (IFOF) and corticospinal tract, with additional associations in the right arcuate and uncinate fasciculi for musical pitch and prosody measures. In prognostic analyses, integrity of the IFOF and uncinate fasciculus at < 3 weeks significantly predicted 3‑month performance across all measured pitch parameters.

**Conclusion:**

These results point to overlapping behavioral and neural associations related to pitch‐based perception, underscoring its importance in both music and speech domains.

## Graphical abstract

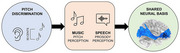

## 1 Introduction

Pitch refers to the subjective auditory sensation of the highness or lowness of a sound based on its perceived periodicity, represented by the fundamental frequency (F0). Accurate pitch perception enables recognition of melodic contours, tonal hierarchies, and harmonic relationships, forming the basis for the processing of musical structure and our aesthetic experience (Mehr 2025; Schellenberg et al. 2000). Similarly, in spoken language, variations in F0 provide prosodic cues that indicate word stress, sentence intonation, and emotional tone. These pitch‐based cues are essential for parsing linguistic structure and interpreting affective meaning, referred to as linguistic prosody and affective prosody, respectively (Arvaniti 2020; Monrad‐Krohn 1947). Together, these observations suggest that pitch represents an important organizing dimension of acoustic information processing in both music and speech, contributing to shared perceptual constraints that support human communication across domains.

A central question in auditory neuroscience is whether pitch‐based processing in music and speech relies on shared auditory–perceptual processing across early and intermediate stages or reflects partially distinct, domain‑specific processes (Patel 2003; Perrachione et al. 2013; Zatorre and Baum 2012). Behavioral evidence offers mixed support: while pitch discrimination has been proposed as a general auditory capacity rooted in early encoding of F0, several studies indicate that music and speech place different demands on pitch resolution, leading to domain‑specific advantages or dissociations (Bent et al. 2006; Ozaki et al. 2024; Shahin 2011; Tillmann 2014). For example, the processing of musical interval relationships used in scales requires more fine‐grained, accurate encoding of pitch, whereas more coarse‐grained processing of pitch suffices for perception of speech contours crucial, for example, in prosody (Zatorre and Baum 2012). Associations between the domains have also been observed, with tone‑language speakers, musicians, and individuals with congenital amusia showing converging patterns of pitch‑related strengths or impairments across music and speech (Oechslin et al. 2010; Shao et al. 2020; Sun et al. 2017; Tillmann et al. 2023; Toh et al. 2023). Neuroimaging findings parallel these behavioral observations, revealing partially overlapping but non‑identical networks for music and prosody within the right hemisphere (Escoffier et al. 2013; Merrill et al. 2012; Peretz et al. 2015; Sihvonen et al. 2019; Tillmann et al. 2023), with the inferior fronto‑occipital fasciculus (IFOF), a ventral pathway linking temporal regions responsible for acoustic analysis with frontal areas involved in higher‐level integration and interpretation, emerging as a putative key pathway supporting pitch perception across domains (Sihvonen et al. 2022). Despite this converging evidence, the extent to which pitch discrimination in music and speech relies on shared versus domain‑specific neural mechanisms remains unresolved. In this context, shared effects may reflect overlapping contributions from early auditory encoding of F0 and subsequent perceptual integration of pitch information, rather than fully shared higher‑order representations.

To date, relatively few studies have examined music‐ and speech‐related pitch‐based tasks within the same individuals and combined structural neuroimaging with behavioral analyses (Norman‐Haignere et al. 2015; Sankaran et al. 2024). Stroke provides a lesion‐based framework that constrains interpretations of structure‐behavior relationships (Rorden and Karnath 2004). Lesions can selectively disrupt components of auditory and language networks, allowing researchers to examine associations between behavioral performance and structural connectivity. Yet, few studies have systematically investigated whether pitch discrimination predicts musical and prosodic abilities in stroke patients, or whether these functions converge on shared white‐matter pathways.

The present study addresses these gaps by combining behavioral and neuroimaging approaches. We assessed pitch discrimination across a range of deviation magnitudes (25–400 cents), musical pitch perception, and both linguistic and affective prosody perception in a cohort of 39 stroke patients. Behavioral testing was conducted at early subacute (within 3 weeks) and late subacute (3 months) poststroke stages to evaluate whether associations among pitch discrimination, musical pitch, and prosody perception remain stable across recovery, thereby capturing domain‑general associations rather than transient post‑stroke effects. Diffusion MRI correlational tractography was used to identify white matter tracts correlating with these auditory measures cross‐sectionally at the early subacute stage. Moreover, the prognostic analyses examining whether early subacute white matter integrity predicted late subacute pitch‑related outcomes were carried out. We hypothesized that pitch discrimination would correlate with musical pitch and prosody perception at both time points, and that shared white matter connectivity, particularly within the right ventral stream, would support these functions.

## 2 Materials and Methods

### 2.1 Subjects and Study Design

**FIGURE 1:**
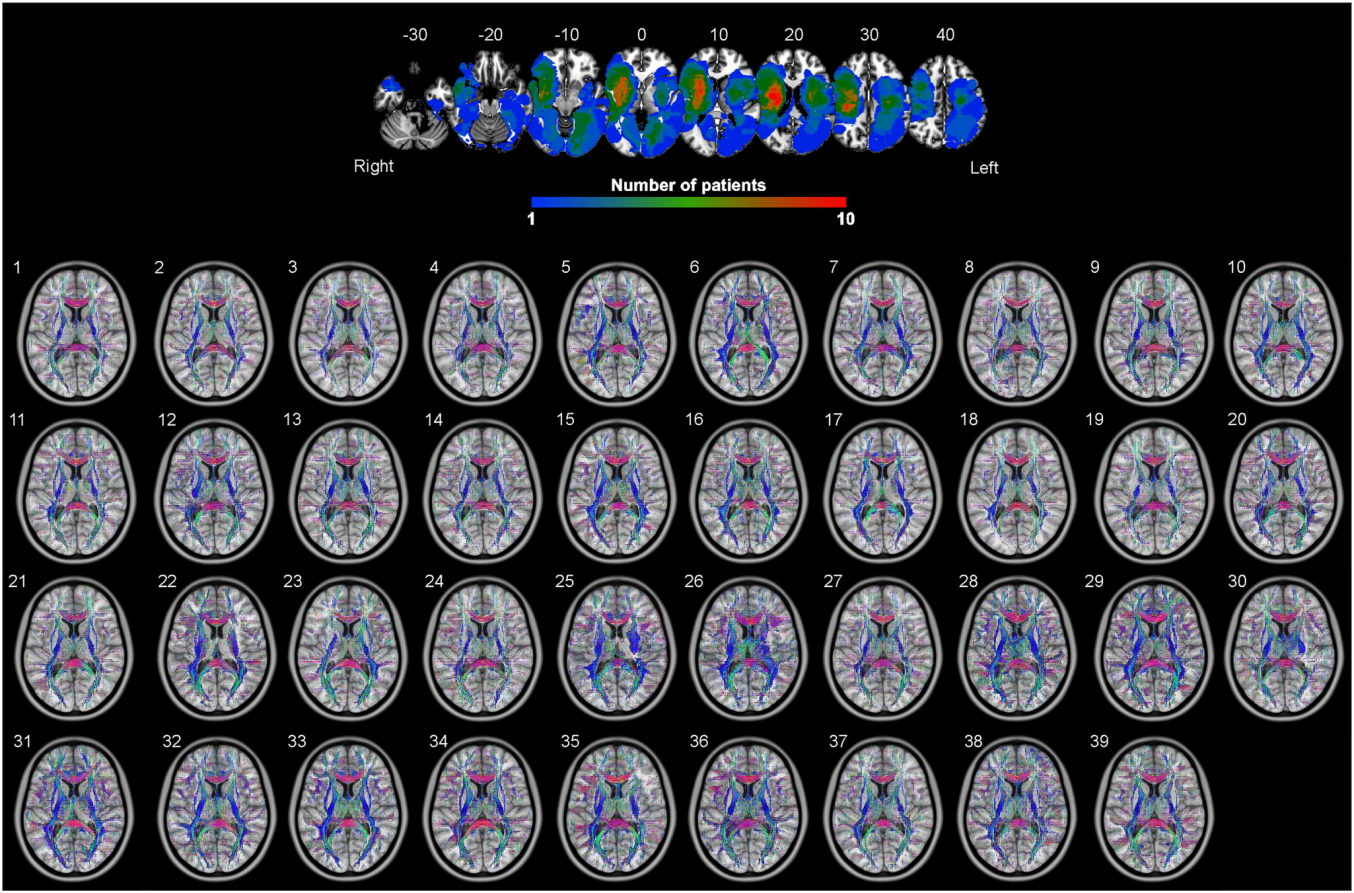
Lesion distribution and orientation distribution function (ODF) registration to MNI space across the whole sample (*N* = 39). The lesion overlap map illustrates the spatial distribution of stroke lesions across participants, with warmer colors indicating increasing overlap in accordance with the color bar. Representative slices also show the alignment of individual ODFs following Q‑space diffeomorphic reconstruction, demonstrating successful registration to MNI space.

Thirty‐nine patients (17 women, 22 men; mean age 56.5 years, SD 14.7) hospitalized for acute stroke were recruited between 2013 and 2015 at the Neurocenter of Turku University Hospital (see Table 1 and Figure 1). Inclusion criteria comprised acute unilateral stroke, right‑handedness, age under 80 years, ability to communicate in Finnish, residence in Southwest Finland, capacity to cooperate with testing, and normal hearing confirmed through clinical screening, absence of self‑reported difficulties, and adequate functional hearing during communication and practice trials. Patients with a history of neurological or psychiatric disorders or substance abuse were excluded. All participants provided written informed consent in accordance with the Declaration of Helsinki, and the study protocol was approved by the Ethics Committee of the Hospital District of Southwest Finland (85/180/2011). MRI and behavioral assessments were conducted within 3 weeks of stroke onset (mean 12.1 days, SD 5.5 days) and again at 3 months poststroke (mean 100 days, SD 8.8 days) to evaluate the stability of findings across time. All patients received standard stroke care and rehabilitation. None were professional musicians.

**TABLE 1:** Baseline demographic and clinical characteristics of the patients (*N* = 39).

Demographic information	
Age (years)	56.5 (14.7)
Sex (female/male)	17/22
Education (years)	14.0 (4.1)
Musical training (years)	0.5 (1.3)

Clinical information	
Lesion size (dL)	0.5 (0.5)
Lesion laterality (left/right)	21/18
MBEA Scale score %	73.8 (18.8)
Pitch discrimination (total) score %	86.3 (11.5)
Language prosody score %	67.1 (13.8)
Affective prosody score %	54.7 (16.5)

*Note*: Data are mean (SD) unless otherwise stated.

Abbreviation: MBEA = Montreal Battery of Evaluation of Amusia.

### 2.2 Behavioral Assessment

Prosody perception was evaluated with two validated tasks. In the linguistic prosody task (Hausen et al. 2013), patients heard 30 verbal utterances differing in their word‑stress patterns (Finnish compound word vs. two‐word phrase, such as “KISsankello” vs. “KISsan KELlo”; in English, a comparable example would be “BLUEbell” referring to the name of a flower and “BLUE BELL” referring to a colored bell) and selected the matching picture from two options. In the affective prosody task (Leinonen et al. 1997), patients heard 96 one‑word utterances (“Saara”, a Finnish female first name) spoken with happy, sad, angry, afraid, surprised, or neutral intonation and chose the corresponding emotion from six on‑screen options.

Musical pitch perception was measured using the scale subtest of the shortened version (Särkämö et al. 2009) of the Montreal Battery of Evaluation of Amusia (MBEA) (Peretz et al. 2003). In the Scale subtest, patients were presented with 14 pairs of piano melodies, half of which were identical and half of which contained an out‑of‑scale pitch change in the second melody, and they judged, on each trial, whether the target and comparison melodies were the same or not.

In the pitch discrimination test (adapted from Hyde and Peretz 2004), a standard tone of 1047 Hz (corresponding to C6) served as the reference stimulus, while deviant tones were generated by altering the frequency either downward (low change) or upward (high change) relative to the standard. Low‐frequency changes included tones of 1032, 1016 Hz, 985, 919, and 775 Hz, corresponding to 25, 50, 100, 200, and 400 cents, respectively. High‐frequency changes included tones of 1062, 1078, 1109, 1175, and 1319 Hz, also corresponding to 25, 50, 100, 200, and 400 cents. Here, 100 cents equals one semitone, the smallest tonal unit in Western music. All tones were mono sine waves sampled at 44.1 kHz with a maximum peak amplitude of −3 dB. The duration of a tone was 100 ms with 10 ms linear ramp at start and end. The experiment consisted of four randomized sequences of 20 trials each, totaling 80 trials. Each sequence contained all stimulus types repeated four times in random order. Within each trial, five auditory stimuli were presented: the first three were always the standard tone, followed by either a deviant tone (one of the ten frequency changes) or another standard tone with equal probability (50%), and finally another standard tone. After the last tone, participants were asked to judge whether the tones in the sequence were all the same or if the sequence contained a pitch change. Stimuli were presented with a stimulus onset asynchrony of 350 ms, and the question appeared 100 ms after the offset of the final tone.

All auditory tasks were presented binaurally via headphones on a laptop computer using Presentation software. In order to minimize potential effects of fatigue and attentional difficulties on task performance, the tasks were performed in a quiet room over multiple shorter testing sessions, if needed, and included regular breaks. Before the tasks, the sound volume was adjusted to a comfortable, clearly audible level. Response time was unlimited, and the next trial began immediately after a response. Correct responses were calculated for the total scores of the linguistic prosody, affective prosody, and MBEA Scale tasks and separately for each deviation magnitude (in cents) and for the overall performance across all conditions (total) in the pitch discrimination task. All behavioral scores at both time points were converted to percentage‐correct values for analysis. Statistical analyses comprised Spearman correlations (two‐tailed) between the behavioral variables at both time points. False discovery rate (FDR) was used to correct for multiple comparisons, and the alpha level was set to 0.05.

### 2.3 MRI Data Acquisition and Preprocessing

Patients were scanned on a 3T Siemens Magnetom Verio scanner at the Department of Radiology of Turku University Hospital. Diffusion MRI scans (TR = 11700 ms, TE = 88 ms, acquisition matrix = 112 × 112, 66 axial slices, voxel size = 2.0 × 2.0 × 2.0 mm^3^) with one non‐diffusion weighted volume and 64 diffusion weighted volumes (*b*‐value = 1000 s/mm^2^) were acquired at the early subacute stage.

Diffusion MRI data were processed using the FMRIB Software Library (FSL v5.0.8, www.fmrib.ox.ac.uk/fsl). First, eddy current distortions and head motions were corrected, followed by gradient matrix rotation using FSL's fdt rotate bvecs. Then, brain extraction was performed using the Brain Extraction Tool. Following preprocessing, diffusion MRI data were reconstructed in Montreal Neurological Institute (MNI) space using Q‐space diffeomorphic reconstruction (QSDR) (Yeh and Tseng 2011) in DSI Studio (http://dsi‐studio.labsolver.org), with a diffusion sampling length ratio of 1.25 to generate spin distribution functions (SDFs) in normalized diffusion space (Yeh et al. 2010). During reconstruction, a white matter mask was applied within the QSDR framework to minimize lesion‑induced distortions and ensure accurate spatial normalization to MNI space, consistent with the original implementation of connectometry in stroke populations (Yeh, Tang, et al. 2013). Outlier detection procedure was applied during preprocessing, and no data were flagged as low‑quality outliers. Restricted diffusion was quantified using restricted diffusion imaging (Yeh et al. 2017). Quantitative anisotropy (QA) values were extracted as local connectome fingerprints (Yeh, Vettel, et al. 2016) for subsequent connectometry analyses (Yeh, Badre, et al. 2016).

### 2.4 Correlation Tractography

Correlational tractography (Yeh et al. 2021) using connectometry was performed to identify white matter pathways in which QA was associated with four behavioral measures (linguistic prosody, affective prosody, MBEA Scale, and pitch discrimination), based on preprocessed early subacute diffusion MRI data. Connectometry does not assume normality of diffusion metrics or spatially homogeneous effects along entire tracts; instead, it evaluates local, segment‐wise structure‐behavior associations. Importantly, correlational tractography evaluates associations between inter‑individual variation in QA and behavioral performance at each local fiber segment across the full sample, rather than contrasting lesioned versus non‑lesioned tissue within individuals. Statistical significance was assessed using non‑parametric Spearman correlations and permutation‑based inference, in which the length of coherently associated fiber segments was compared against a null distribution generated from randomized permutations, with FDR correction applied to control for multiple comparisons. In addition, four predictive analyses were conducted to examine whether early subacute white matter integrity predicted late subacute pitch‑related outcomes. For all analyses, statistical inference was performed using permutation testing (Yeh, Badre, et al. 2016). Fiber tracking employed a *T*‑score threshold of 2.5 (corresponding to an effect size of 0.38) (Yeh, Verstynen, et al. 2013). The tracks were filtered by topology‐informed pruning (Yeh et al. 2019) with 24 iterations. An FDR threshold of 0.05 was used to select tracks. To estimate the FDR, a total of 1000 randomized permutations were applied to the group label to obtain the null distribution of the track length.

In addition to the correlational tractography analyses, we examined the relationship between lesion volume at the early subacute stage and all assessed pitch‑related behavioral measures using two‐tailed Spearman correlations, in order to evaluate the extent to which lesion volume contributed to variability in these measures within the stroke population. All resulting *p*‐values were subsequently corrected for multiple comparisons using the FDR procedure. Then, as a sensitivity analysis, both cross‑sectional and prognostic connectometry analyses were repeated with age and lesion volume included as covariates within the connectometry model (see Supporting Information).

## 3 Results

### 3.1 Behavioral Associations

At the early subacute stage, pitch discrimination (total) score demonstrated significant correlations with all target variables: MBEA Scale (*r*_s_ = 0.730, *p*_FDR_ < 0.001), linguistic prosody (*r*_s_ = 0.594, *p*_FDR_ < 0.001), and affective prosody (*r*_s_ = 0.629, *p*_FDR_ < 0.001).

Similar consistent statistically significant associations were observed at the late subacute stage: pitch discrimination (total) score correlated with MBEA Scale (*r*_s_ = 0.782, *p*_FDR_ < 0.001), linguistic prosody (*r*_s_ = 0.566, *p*_FDR_ < 0.001), and affective prosody (*r*_s_ = 0.673, *p*_FDR_ < 0.001).

Next, to explore which deviation magnitude was most strongly associated with other variables of interest, pitch discrimination scores for each deviation level (cent) were analyzed separately and correlated with target variables. The 400‐cent score was excluded from the analyses because all patients scored 100% correct. Out of the individual cent‐level scores, the 50‐cent score showed the most consistent and strongest correlations with all target variables (Table 2): MBEA Scale (*r*_s_ = 0.569, *p*_FDR_ < 0.001), linguistic prosody (*r*_s_ = 0.494, *p*_FDR_ < 0.009), and affective prosody (*r*_s_ = 0.693, *p*_FDR_ < 0.001).

**TABLE 2:** Correlations between pitch discrimination (cent levels) and target variables. Two‑tailed Spearman correlations were computed, and multiple comparisons were controlled using a false discovery rate threshold of *p* < 0.05.

	25‐cents % score	50‐cents % score	100‐cents % score	200‐cents % score
**MBEA Scale % score**	*r*_s_ = 0.265 *p*_FDR_ = 0.164	*r*_s_ = 0.569 *p*_FDR_ < 0.001	*r*_s_ = 0.564 *p*_FDR_ < 0.001	*r*_s_ = 0.455 *p*_FDR_ = 0.015
**Linguistic prosody % score**	*r*_s_ = 0.556 *p*_FDR_ = 0.002	*r*_s_ = 0.494 *p*_FDR_ = 0.009	*r*_s_ = 0.370 *p*_FDR_ = 0.055	*r*_s_ = 0.190 *p*_FDR_ = 0.307
**Affective prosody % score**	*r*_s_ = 0.552 *p*_FDR_ = 0.002	*r*_s_ = 0.693 *p*_FDR_ < 0.001	*r*_s_ = 0.488 *p*_FDR_ = 0.009	*r*_s_ = 0.283 *p*_FDR_ = 0.148

### 3.2 Neural Associations

The relationship between lesion volume at the early subacute stage and all pitch‑related behavioral measures was examined. None of these correlations reached statistical significance, indicating that lesion volume did not account for meaningful variance in pitch discrimination, musical pitch perception, or prosody perception (*p*_FDR_ = 0.068–0.073).

**FIGURE 2:**
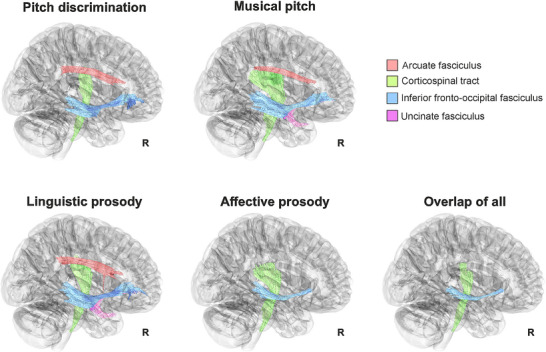
White matter tracts supporting pitch discrimination, musical pitch, and prosody perception. Significant early subacute stage cross‐sectional correlational tractography results (*T* = 2.5, permutations = 1000) showing structural pathways positively associated (FDR < 0.05) with studied behavioral variables and overlap of all findings. R  =  right.

The four calculated early subacute cross‐sectional correlational tractography analyses revealed overlapping white matter pathways supporting pitch discrimination (total), musical pitch perception, and both linguistic and affective prosody perception (Figure 2, Table 3): better performance in all four variables was associated with higher QA values in the right IFOF and corticospinal tract. Moreover, higher MBEA Scale, linguistic prosody, and affective prosody scores were associated with greater QA values in the right arcuate fasciculus, and higher MBEA Scale and linguistic prosody scores were additionally associated with greater QA values in the right uncinate fasciculus.

**TABLE 3:** Significant tract segments associated with pitch discrimination, musical pitch, and prosody perception at the early subacute stage, and predictive of performance at the late subacute stage.

	*p*_FDR_‐value	Segment volume	Segment QA_Mean_
Early subacute stage			
Pitch discrimination			
Right arcuate fasciculus	< 0.001	1943 mm^3^	0.24
Right IFOF	< 0.001	3527 mm^3^	0.23
Right corticospinal tract	< 0.001	3535 mm^3^	0.36
Musical pitch			
Right arcuate fasciculus	< 0.001	1385 mm^3^	0.24
Right IFOF	< 0.001	6426 mm^3^	0.34
Right corticospinal tract	< 0.001	4173 mm^3^	0.24
Right uncinate fasciculus	< 0.001	1254 mm^3^	0.21
Linguistic prosody			
Right arcuate fasciculus	< 0.001	2585 mm^3^	0.24
Right IFOF	< 0.001	3804 mm^3^	0.24
Right corticospinal tract	< 0.001	4478 mm^3^	0.36
Right uncinate fasciculus	< 0.001	1404 mm^3^	0.20
Affective prosody			
Right IFOF	< 0.001	1227 mm^3^	0.25
Right corticospinal tract	< 0.001	4212 mm^3^	0.34
Predictive analyses			
Pitch discrimination			
Right arcuate fasciculus	< 0.001	6150 mm^3^	0.23
Right IFOF	< 0.001	9734 mm^3^	0.23
Right corticospinal tract	< 0.001	9135 mm^3^	0.33
Right uncinate fasciculus	< 0.001	4180 mm^3^	0.19
Right corticostriatal tract	< 0.001	6452 mm^3^	0.24
Musical pitch			
Right IFOF	< 0.001	1006 mm^3^	0.22
Right uncinate fasciculus	< 0.001	4782 mm^3^	0.24
Linguistic prosody			
Right arcuate fasciculus	< 0.001	1988 mm^3^	0.24
Right IFOF	< 0.001	2712 mm^3^	0.24
Right corticospinal tract	< 0.001	3198 mm^3^	0.37
Right uncinate fasciculus	< 0.001	616 mm^3^	0.23
Affective prosody			
Right arcuate fasciculus	< 0.001	2792 mm^3^	0.24
Right IFOF	< 0.001	4519 mm^3^	0.24
Right corticospinal tract	< 0.001	6537 mm^3^	0.33
Right uncinate fasciculus	< 0.001	1620 mm^3^	0.21

In cross‑sectional analyses, inclusion of age and lesion volume as covariates yielded a broadly comparable pattern of structure–behavior associations. However, the right IFOF association with affective prosody at the early subacute stage did not retain statistical significance (Supporting Information, Figure S1).

**FIGURE 3:**
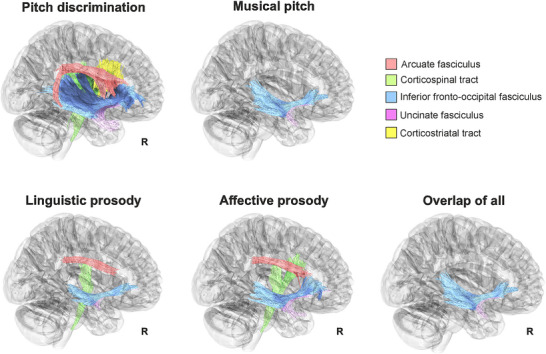
White matter tracts predicting pitch discrimination, musical pitch, and prosody perception at the late subacute stage. Correlational tractography results (*T* = 2.5, permutations = 1000) showing early subacute stage structural pathways positively predicting late subacute stage performance (FDR < 0.05) in studied behavioral variables and overlap of all findings. R  =  right.

Similar findings were present in prognostic analyses (Figure 3, Table 3): the early subacute stage integrity of the IFOF and uncinate fasciculus significantly predicted 3‑month performance across all measured pitch parameters. Moreover, higher MBEA Scale, linguistic prosody, and affective prosody scores were predicted by greater QA values in the right arcuate fasciculus and corticospinal tract, and higher MBEA Scale was additionally predicted by greater QA values in the right corticostriatal tract.

Similarly, in prognostic analyses, inclusion of age and lesion volume yielded a broadly comparable pattern of structure‐behavior associations. However, the association between the right IFOF and linguistic prosody did not retain statistical significance after covariate adjustment (Supporting Information, Figure S1).

## 4 Discussion

This study investigated whether pitch discrimination is associated with musical and speech‑related pitch perception and whether these domains engage common white matter circuitry. Using behavioral assessments and diffusion MRI correlational tractography in a cohort of stroke patients, we demonstrate that pitch discrimination is linked to both musical pitch perception and prosody perception, supporting partially overlapping behavioral and neural correlates. Our findings also suggest that these functions share common neural pathways within the right ventral stream but depend on partially distinct neural pathways to fulfill domain‐specific demands.

The results indicate that better pitch discrimination was associated with better performance in musical pitch, linguistic prosody, and affective prosody tasks at both early and late subacute stages. The consistency of these relationships across time supports the view that these behavioral links reflect robust, domain‑general auditory mechanisms rather than transient effects tied to the immediate post‑lesional state. This convergence aligns with theories proposing a general‐purpose auditory capacity rooted in early neural encoding of F0 (Tillmann 2014). However, the degree of correlation varied across domains, suggesting that while pitch discrimination is a common prerequisite, music and speech impose different processing demands. Musical pitch perception often requires fine‐grained resolution to detect small intervallic differences, whereas prosody relies on broader contours or categorical distinctions, particularly in tone languages (Bent et al. 2006). In congenital amusia (Hyde and Peretz 2004; Tillmann et al. 2023), the fine‐grained pitch perception deficit has been observed for small pitch changes (i.e., 25 and 50 cents). Similarly, the current results indicated that a 50‐cent score showed the most consistent and strongest correlations with all pitch‐based target variables, supporting this finding from congenital amusia. Fine‑grained pitch sensitivity in this range is important for prosodic comprehension, including intonation, stress, and emotional tone (Lolli et al. 2015), possibly contributing to difficulties in perceiving prosodic cues after stroke.

Correlational tractography analyses revealed that all the pitch perception domains analyzed were associated with an overlapping structural circuit within the right hemisphere ventral stream, particularly the IFOF. This tract links temporal regions involved in acoustic analysis with frontal areas supporting higher‐order integration and has been linked to both music perception (Dohn et al. 2015; Sihvonen et al. 2017, 2022) and prosody perception (Frühholz et al. 2015; Jackson et al. 2025; Sammler et al. 2015; Sihvonen et al. 2022). The involvement of the IFOF underscores the importance of distributed networks for pitch‐mediated communication across domains. Nevertheless, the absence of complete overlap between tracts associated with pitch discrimination and the musical and prosodic tasks suggests that domain‐specific adaptations exist within this shared architecture, including additional involvement of the right arcuate fasciculus and uncinate fasciculus in music and prosody perception. This right‑lateralized pattern is consistent with previous lesion and neuroimaging evidence showing that pitch‑based auditory functions, including prosodic contour perception, are predominantly supported by the right ventral stream (Dohn et al. 2015; Sammler et al. 2015; Sierpowska et al. 2025; Sihvonen et al. 2017; Vaquero et al. 2021). The observed association between corticospinal tract integrity and performance on the pitch‐related tasks is not straightforward and is unlikely to reflect a primary role of this pathway in pitch or prosodic processing. One possibility is that this association relates indirectly to increased cognitive effort, predictive processing during active listening, or broader auditory‐motor and affective engagement (Antunes and Malmierca 2021; Furukawa et al. 2017; Giovannelli et al. 2013). However, this finding should be interpreted with caution and is best regarded as exploratory, as it may also reflect non‐specific lesion effects or generalized motor‑related variance rather than task‑specific auditory‐motor coupling. Overall, our findings support a hybrid model in which pitch perception relies on common perceptual and neural mechanisms but diverges at higher levels of integration to accommodate the distinct structural and functional constraints of music and speech.

Importantly, sensitivity analyses including age and lesion volume indicated that not all IFOF‐related associations remained statistically significant across domains. In particular, the associations with affective prosody at the early subacute stage and linguistic prosody in the prognostic analyses were attenuated after covariate adjustment. This suggests that, while the IFOF constitutes a central component of the observed network, its contribution is not uniformly robust across all pitch‐related domains when accounting for clinical variability in the current sample. Given the modest sample size relative to the inclusion of multiple covariates, these attenuated effects should be interpreted with some caution. Accordingly, the present findings are best understood as reflecting substantial but not complete overlap in the neural substrates underlying pitch‐based processing in music and speech.

By examining the behavioral consequences of focal brain lesions, the present findings provide lesion‑based evidence linking right hemisphere ventral white matter pathways to pitch‑based aspects of auditory communication. Both prosody and music perception depend on the encoding, integration, and evaluation of pitch sequences and temporal patterns within rapidly unfolding acoustic signals. Within contemporary models of auditory processing, these demands are thought to engage partially overlapping mechanisms along the ventral auditory stream that support the integration of acoustic information with higher‑order interpretive processes (Rauschecker and Scott 2009). From this perspective, disrupted integrity of the right IFOF and its frontal termination zones may be associated with joint difficulties in musical and prosodic perception, as observed in the present cohort. More generally, the observed relationships between tract integrity and behavioral performance underscore the sensitivity of pitch‑based processing to disruptions in long‑range connectivity and support a distributed network account of auditory–linguistic integration. These findings extend prior work by situating musical and prosodic processing within shared ventral‑stream connectivity while allowing for domain‑specific modulation within this broader network architecture (Sammler et al. 2015; Sihvonen et al. 2022).

The strong link between pitch discrimination and prosody comprehension has practical implications for rehabilitation. Prosodic deficits can occur after stroke and can significantly impair social communication (Guo et al. 2021; O'Connell et al. 2024). Our findings suggest that assessing pitch discrimination may help identify patients at risk for broader communicative difficulties. Furthermore, interventions targeting pitch perception—such as music‐based interventions—could potentially enhance prosodic skills (Neves et al. 2022; Patel 2014), although the degree of transfer remains to be established in stroke patients. Future research should explore whether training paradigms that explicitly engage both domains yield greater benefits than domain‐specific approaches.

Several limitations warrant consideration. First, the sample size and lesion heterogeneity constrain the generalizability of our findings. Second, while diffusion MRI provides valuable insights into structural connectivity, it cannot fully capture functional dynamics or compensatory mechanisms. Third, our tasks focused on pitch‐based aspects of music and prosody, leaving other dimensions (e.g., rhythm, timbre) unexplored. Temporal and rhythmic processing are also fundamental to both music and speech, but these dimensions were outside the scope of the present study and were not assessed behaviorally or neurally. Thus, the current findings address pitch‐based processing specifically and should not be taken to represent a comprehensive account of shared auditory mechanisms. Fourth, pitch discrimination was assessed using brief sine tones, which have limited ecological validity relative to real‑world music and speech. Accordingly, the findings should be interpreted as reflecting basic pitch sensitivity rather than naturalistic auditory processing. Fifth, allowing unlimited response time may have attenuated the detection of subtle performance deficits. Future studies should adopt longitudinal designs to track recovery trajectories and incorporate functional imaging to examine network reorganization. Comparative work involving tone‐language speakers and musicians could further clarify the interplay between experience‐dependent plasticity and domain‐specific specialization. Furthermore, future studies using larger cohorts would be better positioned to model the effects of clinical variables (e.g., age, lesion size) on pitch‑based perception more comprehensively, thereby reducing residual confounding. The longitudinal analyses in the present study were designed to test whether early subacute white matter integrity predicts later behavioral performance, rather than to characterize recovery trajectories over time. Future studies with additional time points will be important for directly modeling recovery processes alongside prognostic effects.

In summary, this study demonstrates that pitch discrimination is a critical component of both musical and prosodic processing, supported by strongly overlapping but not identical neural networks. These findings advance our understanding of auditory communication and highlight the importance of considering both shared and specialized mechanisms in models of pitch perception. By integrating behavioral and neuroimaging evidence, we provide a foundation for future research aimed at refining theoretical frameworks and developing targeted interventions for individuals with communicative impairments.

## Author Contributions

**Aleksi J. Sihvonen**: conceptualization, methodology, data curation, investigation, formal analysis, funding acquisition, visualization, writing – original draft, writing – review and editing. **Teppo Särkämö**: writing – original draft, writing – review and editing, funding acquisition, conceptualization, methodology, supervision, project administration. **Tommi Makkonen**: conceptualization, methodology, software, formal analysis, resources, writing – review and editing, writing – original draft.

## Funding

This study was supported by external funding from the Research Council of Finland (grants 257077, 277693, 299044, 368008, 346211, 364220), Finnish Cultural Foundation (grant 191230), Orion Research Foundation sr, and Signe and Ane Gyllenberg Foundation.

## Ethics Statement

The study protocol was approved by the Ethics Committee of the Hospital District of Southwest Finland (85/180/2011).

## Consent

All participants gave informed written consent in accordance with the Declaration of Helsinki.

## Conflicts of Interest

The authors declare no conflicts of interest.

## Supporting information




**Supporting Information**: brb371512‐sup‐0001‐SuppMat.docx

## Data Availability

Anonymized data reported in this manuscript are available from the corresponding author upon reasonable request and subject to approval by the appropriate regulatory committees and officials.

## References

[brb371512-bib-0001] Antunes, F. M. , and M. S. Malmierca . 2021. “Corticothalamic Pathways in Auditory Processing: Recent Advances and Insights From Other Sensory Systems.” Frontiers in Neural Circuits 15: 721186. 10.3389/FNCIR.2021.721186. 34489648 PMC8418311

[brb371512-bib-0002] Arvaniti, A. 2020. “The Phonetics of Prosody.” In Oxford Research Encyclopedia of Linguistics, edited by M. Aronoff . Oxford Academic. 10.1093/ACREFORE/9780199384655.013.411.

[brb371512-bib-0003] Bent, T. , A. R. Bradlow , and B. A. Wright . 2006. “The Influence of Linguistic Experience on the Cognitive Processing of Pitch in Speech and Nonspeech Sounds.” Journal of Experimental Psychology: Human Perception and Performance 32, no. 1: 97–103. 10.1037/0096-1523.32.1.97. 16478329

[brb371512-bib-0004] Dohn, A. , E. A. Garza‐Villarreal , M. M. Chakravarty , M. Hansen , J. P. Lerch , and P. Vuust . 2015. “Gray‐and White‐Matter Anatomy of Absolute Pitch Possessors.” Cerebral Cortex 25, no. 5: 1379–1388. 10.1093/cercor/bht334. 24304583

[brb371512-bib-0005] Escoffier, N. , J. Zhong , A. Schirmer , and A. Qiu . 2013. “Emotional Expressions in Voice and Music: Same Code, Same Effect?” Human Brain Mapping 34, no. 8: 1796–1810. 10.1002/HBM.22029. 22505222 PMC6870024

[brb371512-bib-0006] Frühholz, S. , M. Gschwind , and D. Grandjean . 2015. “Bilateral Dorsal and Ventral Fiber Pathways for the Processing of Affective Prosody Identified by Probabilistic Fiber Tracking.” Neuroimage 109: 27–34. 10.1016/j.neuroimage.2015.01.016. 25583613

[brb371512-bib-0007] Furukawa, Y. , K. Uehara , and S. Furuya . 2017. “Expertise‐Dependent Motor Somatotopy of Music Perception.” Neuroscience Letters 650: 97–102. 10.1016/j.neulet.2017.04.033. 28435044

[brb371512-bib-0008] Giovannelli, F. , C. Banfi , A. Borgheresi , et al. 2013. “The Effect of Music on Corticospinal Excitability Is Related to the Perceived Emotion: A Transcranial Magnetic Stimulation Study.” Cortex 49, no. 3: 702–710. 10.1016/j.cortex.2012.01.013. 22405960

[brb371512-bib-0009] Guo, C. , C. Stretz , J. R. Anderson , et al. 2021. “Psychiatric Sequelae of Stroke Affecting the Non‐Dominant Cerebral Hemisphere.” Journal of the Neurological Sciences 430: 120007. 10.1016/j.jns.2021.120007. 34624794

[brb371512-bib-0010] Hausen, M. , R. Torppa , V. R. Salmela , M. Vainio , and T. Särkämö . 2013. “Music and Speech Prosody: A Common Rhythm.” Frontiers in Psychology 4: 566. 10.3389/fpsyg.2013.00566. 24032022 PMC3759063

[brb371512-bib-0011] Hyde, K. L. , and I. Peretz . 2004. “Brains That Are Out of Tune but in Time.” Psychological Science 15, no. 5: 356–360. 10.1111/j.0956-7976.2004.00683.x. 15102148

[brb371512-bib-0012] Jackson, M. S. , Y. Uchida , S. M. Sheppard , et al. 2025. “Elucidating White Matter Contributions to the Cognitive Architecture of Affective Prosody Recognition: Evidence From Right Hemisphere Stroke.” Brain Sciences 15, no. 7: 769. 10.3390/BRAINSCI15070769. 40722359 PMC12293220

[brb371512-bib-0013] Leinonen, L. , T. Hiltunen , I. Linnankoski , and M.‐L. Laakso . 1997. “Expression of Emotional–Motivational Connotations With a One‐Word Utterance.” Journal of the Acoustical Society of America 102, no. 3: 1853–1863. 10.1121/1.420109. 9301063

[brb371512-bib-0014] Lolli, S. L. , A. D. Lewenstein , J. Basurto , S. Winnik , and P. Loui . 2015. “Sound Frequency Affects Speech Emotion Perception: Results From Congenital Amusia.” Frontiers in Psychology 6: 1340. 10.3389/fpsyg.2015.01340. 26441718 PMC4561757

[brb371512-bib-0015] Mehr, S. A. 2025. “Core Systems of Music Perception.” Trends in Cognitive Sciences 29, no. 8: 763–777. 10.1016/j.tics.2025.05.013. 40517090 PMC12328076

[brb371512-bib-0016] Merrill, J. , D. Sammler , M. Bangert , et al. 2012. “Perception of Words and Pitch Patterns in Song and Speech.” Frontiers in Psychology 3: 76. 10.3389/FPSYG.2012.00076. 22457659 PMC3307374

[brb371512-bib-0017] Monrad‐Krohn, G. H. 1947. “The Prosodic Quality of Speech and Its Disorders: (A Brief Survey From a Neurologist's Point of View).” Acta Psychiatrica Scandinavica 22, no. 3–4: 255–269. 10.1111/j.1600-0447.1947.tb08246.x.

[brb371512-bib-0018] Neves, L. , A. I. Correia , S. L. Castro , D. Martins , and C. F. Lima . 2022. “Does Music Training Enhance Auditory and Linguistic Processing? A Systematic Review and Meta‐Analysis of Behavioral and Brain Evidence.” Neuroscience & Biobehavioral Reviews 140: 104777. 10.1016/J.NEUBIOREV.2022.104777. 35843347

[brb371512-bib-0019] Norman‐Haignere, S. , N. G. Kanwisher , and J. H. McDermott . 2015. “Distinct Cortical Pathways for Music and Speech Revealed by Hypothesis‐Free Voxel Decomposition.” Neuron 88, no. 6: 1281–1296. 10.1016/j.neuron.2015.11.035. 26687225 PMC4740977

[brb371512-bib-0020] O'Connell, K. , A. A. Marsh , and A. Seydell‐Greenwald . 2024. “Right Hemisphere Stroke Is Linked to Reduced Social Connectedness in the UK Biobank Cohort.” Scientific Reports 14, no. 1: 27293. 10.1038/S41598-024-78351-0. 39516519 PMC11549225

[brb371512-bib-0021] Oechslin, M. S. , M. Meyer , and L. Jancke . 2010. “Absolute Pitch—Functional Evidence of Speech‐Relevant Auditory Acuity.” Cerebral Cortex 20, no. 2: 447–455. 10.1093/CERCOR/BHP113. 19592570 PMC2803739

[brb371512-bib-0022] Ozaki, Y. , A. Tierney , P. Q. Pfordresher , et al. 2024. “Globally, Songs and Instrumental Melodies Are Slower and Higher and Use More Stable Pitches Than Speech: A Registered Report.” Science Advances 10, no. 20: eadm9797. 10.1126/SCIADV.ADM9797. 38748798 PMC11095461

[brb371512-bib-0023] Patel, A. D. 2003. “Language, Music, Syntax and the Brain.” Nature Neuroscience 6, no. 7: 674–681. 10.1038/nn1082. 12830158

[brb371512-bib-0024] Patel, A. D. 2014. “Can Nonlinguistic Musical Training Change the Way the Brain Processes Speech? The Expanded OPERA Hypothesis.” Hearing Research 308: 98–108. 10.1016/j.heares.2013.08.011. 24055761

[brb371512-bib-0025] Peretz, I. , A. S. Champod , and K. Hyde . 2003. “Varieties of Musical Disorders: The Montreal Battery of Evaluation of Amusia.” Annals of the New York Academy of Sciences 999: 58–75. 10.1196/annals.1284.006. 14681118

[brb371512-bib-0026] Peretz, I. , D. Vuvan , M. É. Lagrois , and J. L. Armony . 2015. “Neural Overlap in Processing Music and Speech.” Philosophical Transactions of the Royal Society B: Biological Sciences 370, no. 1664: 20140090. 10.1098/rstb.2014.0090. PMC4321131 25646513

[brb371512-bib-0027] Perrachione, T. K. , E. G. Fedorenko , L. Vinke , E. Gibson , and L. C. Dilley . 2013. “Evidence for Shared Cognitive Processing of Pitch in Music and Language.” PLoS ONE 8, no. 8: e73372. 10.1371/journal.pone.0073372. 23977386 PMC3744486

[brb371512-bib-0028] Rauschecker, J. P. , and S. K. Scott . 2009. “Maps and Streams in the Auditory Cortex: Nonhuman Primates Illuminate Human Speech Processing.” Nature Neuroscience 12, no. 6: 718–724. 10.1038/nn.2331. 19471271 PMC2846110

[brb371512-bib-0029] Rorden, C. , and H. O. Karnath . 2004. “Using Human Brain Lesions to Infer Function: A Relic From a Past Era in the fMRI Age?” Nature Reviews Neuroscience 5, no. 10: 812–819. 10.1038/nrn1521. 15378041

[brb371512-bib-0030] Sammler, D. , M. H. Grosbras , A. Anwander , P. E. G. Bestelmeyer , and P. Belin . 2015. “Dorsal and Ventral Pathways for Prosody.” Current Biology 25, no. 23: 3079–3085. 10.1016/j.cub.2015.10.009. 26549262

[brb371512-bib-0031] Sankaran, N. , M. K. Leonard , F. Theunissen , and E. F. Chang . 2024. “Encoding of Melody in the Human Auditory Cortex.” Science Advances 10, no. 7: eadk0010. 10.1126/SCIADV.ADK0010. 38363839 PMC10871532

[brb371512-bib-0032] Särkämö, T. , M. Tervaniemi , S. Soinila , et al. 2009. “Amusia and Cognitive Deficits After Stroke: Is There a Relationship.” Annals of the New York Academy of Sciences 1169: 441–445. 10.1111/j.1749-6632.2009.04765.x. 19673821

[brb371512-bib-0033] Schellenberg, G. E. , A. M. Krysciak , and R. J. Campbell . 2000. “Perceiving Emotion in Melody: Interactive Effects of Pitch and Rhythm.” Music Perception 18, no. 2: 155–171. 10.2307/40285907.

[brb371512-bib-0034] Shahin, A. J. 2011. “Neurophysiological Influence of Musical Training on Speech Perception.” Frontiers in Psychology 2: 126. 10.3389/FPSYG.2011.00126. 21716639 PMC3115576

[brb371512-bib-0035] Shao, J. , L. Wang , and C. Zhang . 2020. “Talker Processing in Mandarin‐Speaking Congenital Amusics.” Journal of Speech, Language, and Hearing Research 63, no. 5: 1361–1375. 10.1044/2020_JSLHR-19-00209. 32343927

[brb371512-bib-0036] Sierpowska, J. , J. Grau‐Sánchez , A. J. Sihvonen , et al. 2025. “Music and Affective Prosody After Surgical Removal of the Right Arcuate Fasciculus: A Case Study.” Annals of the New York Academy of Sciences 1552, no. 1: 314–325. 10.1111/NYAS.70065. 40946316 PMC12576879

[brb371512-bib-0037] Sihvonen, A. J. , P. Ripollés , T. Särkämö , et al. 2017. “Tracting the Neural Basis of Music: Deficient Structural Connectivity Underlying Acquired Amusia.” Cortex 97: 255–273. 10.1016/j.cortex.2017.09.028. 29100660

[brb371512-bib-0038] Sihvonen, A. J. , D. Sammler , P. Ripollés , et al. 2022. “Right Ventral Stream Damage Underlies Both Poststroke Aprosodia and Amusia.” European Journal of Neurology 29, no. 3: 873–882. 10.1111/ene.15148. 34661326

[brb371512-bib-0039] Sihvonen, A. J. , T. Särkämö , A. Rodríguez‐Fornells , P. Ripollés , T. F. Münte , and S. Soinila . 2019. “Neural Architectures of Music—Insights From Acquired Amusia.” Neuroscience & Biobehavioral Reviews 107: 104–114. 10.1016/j.neubiorev.2019.08.023. 31479663

[brb371512-bib-0040] Sun, Y. , X. Lu , H. T. Ho , and W. F. Thompson . 2017. “Pitch Discrimination Associated With Phonological Awareness: Evidence From Congenital Amusia.” Scientific Reports 7, no. 1: 44285. 10.1038/srep44285. 28287166 PMC5347159

[brb371512-bib-0041] Tillmann, B. 2014. “Pitch Processing in Music and Speech.” Acoustics Australia 42, no. 2: 124–130.

[brb371512-bib-0042] Tillmann, B. , J. E. Graves , F. Talamini , et al. 2023. “Auditory Cortex and Beyond: Deficits in Congenital Amusia.” Hearing Research 437: 108855. 10.1016/J.HEARES.2023.108855. 37572645

[brb371512-bib-0043] Toh, X. R. , S. H. Tan , G. Wong , F. Lau , and F. C. K. Wong . 2023. “Enduring Musician Advantage Among Former Musicians in Prosodic Pitch Perception.” Scientific Reports 13, no. 1: 2657. 10.1038/S41598-023-29733-3. 36788323 PMC9929097

[brb371512-bib-0044] Vaquero, L. , N. Ramos‐Escobar , D. Cucurell , et al. 2021. “Arcuate Fasciculus Architecture Is Associated With Individual Differences in Pre‐Attentive Detection of Unpredicted Music Changes.” Neuroimage 229: 117759. 10.1016/j.neuroimage.2021.117759. 33454403

[brb371512-bib-0045] Yeh, F. C. , D. Badre , and T. Verstynen . 2016. “Connectometry: A Statistical Approach Harnessing the Analytical Potential of the Local Connectome.” Neuroimage 125: 162–171. 10.1016/j.neuroimage.2015.10.053. 26499808

[brb371512-bib-0046] Yeh, F. C. , A. Irimia , D. C. D. A. Bastos , and A. J. Golby . 2021. “Tractography Methods and Findings in Brain Tumors and Traumatic Brain Injury.” Neuroimage 245: 118651. 10.1016/j.neuroimage.2021.118651. 34673247 PMC8859988

[brb371512-bib-0047] Yeh, F. C. , L. Liu , T. K. Hitchens , and Y. L. Wu . 2017. “Mapping Immune Cell Infiltration Using Restricted Diffusion MRI.” Magnetic Resonance in Medicine 77, no. 2: 603–612. 10.1002/mrm.26143. 26843524 PMC8052951

[brb371512-bib-0048] Yeh, F. C. , S. Panesar , J. Barrios , et al. 2019. “Automatic Removal of False Connections in Diffusion MRI Tractography Using Topology‐Informed Pruning (TIP).” Neurotherapeutics 16, no. 1: 52–58. 10.1007/s13311-018-0663-y. 30218214 PMC6361061

[brb371512-bib-0049] Yeh, F. C. , P. F. Tang , and W. Y. I. Tseng . 2013. “Diffusion MRI Connectometry Automatically Reveals Affected Fiber Pathways in Individuals With Chronic Stroke.” NeuroImage: Clinical 2, no. 1: 912–921. 10.1016/j.nicl.2013.06.014. 24179842 PMC3777702

[brb371512-bib-0050] Yeh, F. C. , and W. Y. I. Tseng . 2011. “NTU‐90: A High Angular Resolution Brain Atlas Constructed by Q‐Space Diffeomorphic Reconstruction.” Neuroimage 58, no. 1: 91–99. 10.1016/j.neuroimage.2011.06.021. 21704171

[brb371512-bib-0051] Yeh, F. C. , T. D. Verstynen , Y. Wang , J. C. Fernández‐Miranda , and W. Y. I. Tseng . 2013. “Deterministic Diffusion Fiber Tracking Improved by Quantitative Anisotropy.” PLoS ONE 8, no. 11: e80713. 10.1371/journal.pone.0080713. 24348913 PMC3858183

[brb371512-bib-0052] Yeh, F. C. , J. M. Vettel , A. Singh , et al. 2016. “Quantifying Differences and Similarities in Whole‐Brain White Matter Architecture Using Local Connectome Fingerprints.” PLoS Computational Biology 12, no. 11: e1005203. 10.1371/journal.pcbi.1005203. 27846212 PMC5112901

[brb371512-bib-0053] Yeh, F. C. , V. J. Wedeen , and W. Y. I. Tseng . 2010. “Generalized Q‐Sampling Imaging.” IEEE Transactions on Medical Imaging 29, no. 9: 1626–1635. 10.1109/TMI.2010.2045126. 20304721

[brb371512-bib-0054] Zatorre, R. J. , and S. R. Baum . 2012. “Musical Melody and Speech Intonation: Singing a Different Tune.” PLoS Biology 10, no. 7: e1001372. 10.1371/JOURNAL.PBIO.1001372. 22859909 PMC3409119

