# Supplementary material for: Pitch as a Shared Code for Music and Speech: Behavioral and Neural Evidence From Stroke Patients
Source: Brain Behav. 2026 May 29;16(6):e71512. doi: 10.1002/brb3.71512 (PMC13239951; doi:10.1002/brb3.71512)

**SUPPLEMENTARY MATERIAL**

**Pitch as a shared code for music and speech: Behavioural and neural evidence from stroke patients**

Aleksi J. Sihvonen^1,2,3,4,5^, Tommi Makkonen^1,2^, Teppo Särkämö^1,2^

^1^Cognitive Brain Research Unit (CBRU), Department of Psychology, University of Helsinki, Helsinki, Finland.

^2^Centre of Excellence in Music, Mind, Body and Brain, University of Helsinki, Helsinki, Finland.

^3^Department of Neurology, Helsinki University Hospital, Helsinki, Finland.

^4^Queensland Aphasia Research Centre, University of Queensland, Brisbane, Australia.

^5^School of Health and Rehabilitation Sciences, University of Queensland, Brisbane, Australia.

**Correspondence to:** Aleksi J. Sihvonen, Cognitive Brain Research Unit (CBRU), Department of Psychology, University of Helsinki, Haartmaninkatu 3, Helsinki 00290, Finland. Email: [aleksi.sihvonen@helsinki.fi](mailto:aleksi.sihvonen@helsinki.fi)

**Results**

**Neural associations**

To assess the specificity of the observed correlational tractography findings for pitch discrimination (total), musical pitch perception, and linguistic and affective prosody perception, both cross‑sectionally at the early subacute stage and longitudinally in predicting late subacute performance, we controlled all analyses for lesion volume and age (Supplementary Figure 1). These analyses were conducted as robustness checks to evaluate potential stroke lesion‑related effects.

At the early subacute stage, better performance across all four behavioural measures was associated with higher QA values in the right corticospinal tract. In addition, for all measures except affective prosody perception, better performance was related to greater QA values in the right IFOF and the right arcuate fasciculus. Higher QA values in the right uncinate fasciculus were specifically associated with better linguistic prosody perception, whereas greater QA values in the corpus callosum were linked to improved pitch discrimination. Furthermore, higher QA values in the right fornix were associated with better performance in pitch discrimination, musical pitch perception, and affective prosody perception.

In the predictive analyses, higher early subacute QA values in the right corticospinal tract predicted better late subacute performance across all four behavioural measures. Additionally, for all outcomes except linguistic prosody perception, better late subacute performance was predicted by greater early subacute QA values in the right IFOF and right uncinate fasciculus. Higher QA values in the right arcuate fasciculus further predicted improved pitch discrimination and affective prosody perception, while greater QA values in the corticostriatal tract predicted better pitch discrimination and musical pitch perception.

**Supplementary Figure 1. White matter tracts supporting pitch discrimination, musical pitch perception and prosody perception at the early subacute stage and predicting late subacute stage performance.** The figure depicts significant early subacute cross‑sectional correlational tractography results and predictive analysis results showing structural pathways positively associated ((T = 2.5, permutations = 1000, P_FDR_ < 0.05) with the studied behavioural measures, as well as the overlap across analyses. All analyses were controlled for lesion volume and age. R = right.


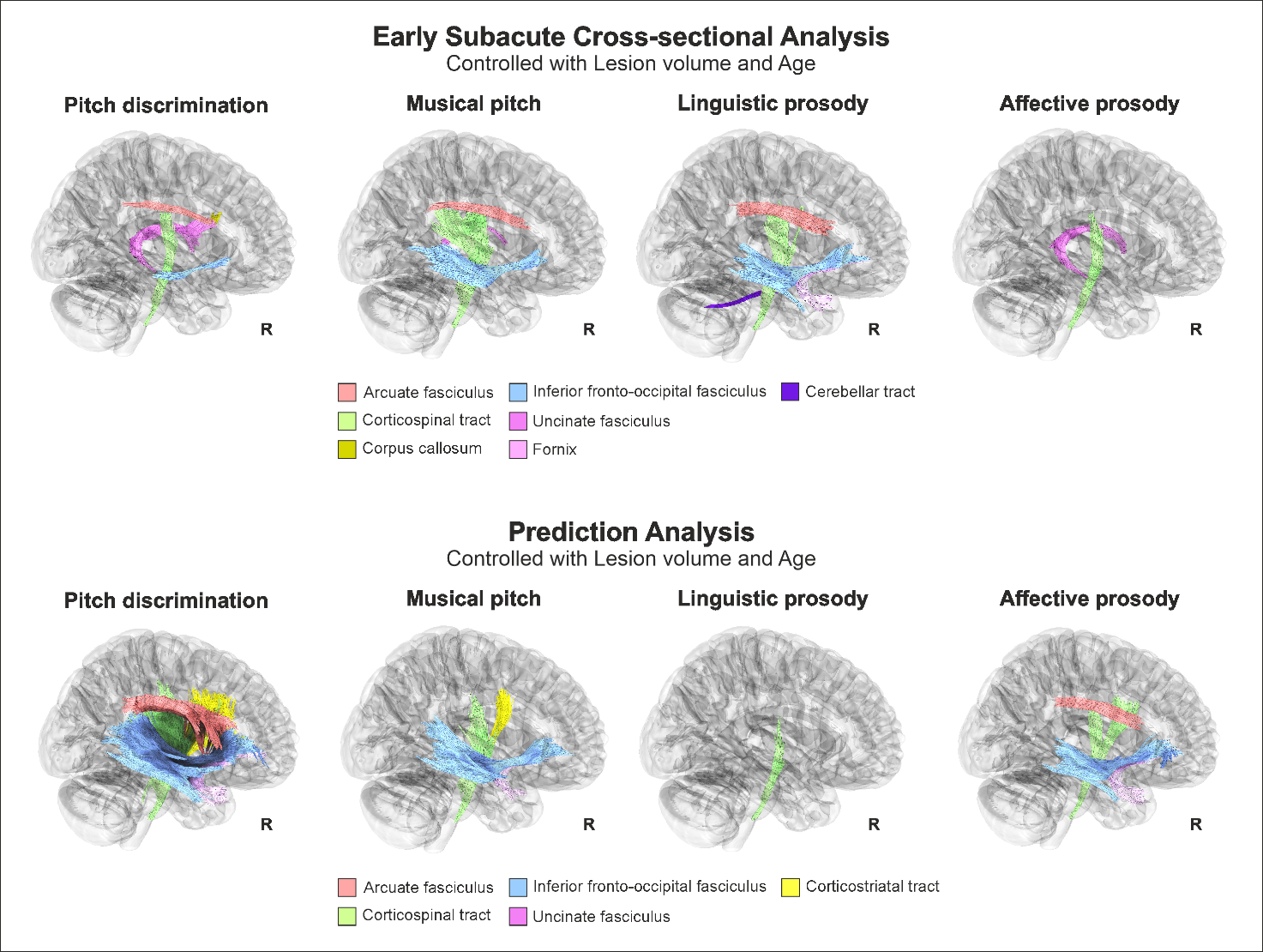

Supplement: Supplementary file 1 — Supporting Information: brb371512‐sup‐0001‐SuppMat.docx [file BRB3-16-e71512-s001.docx]
